# Supplementary figures and images for: Short-term association between ambient air pollution and cardio-respiratory mortality in Rio de Janeiro, Brazil
Source: PLoS One. 2023 Feb 16;18(2):e0281499. doi: 10.1371/journal.pone.0281499 (PMC9934392; doi:10.1371/journal.pone.0281499)

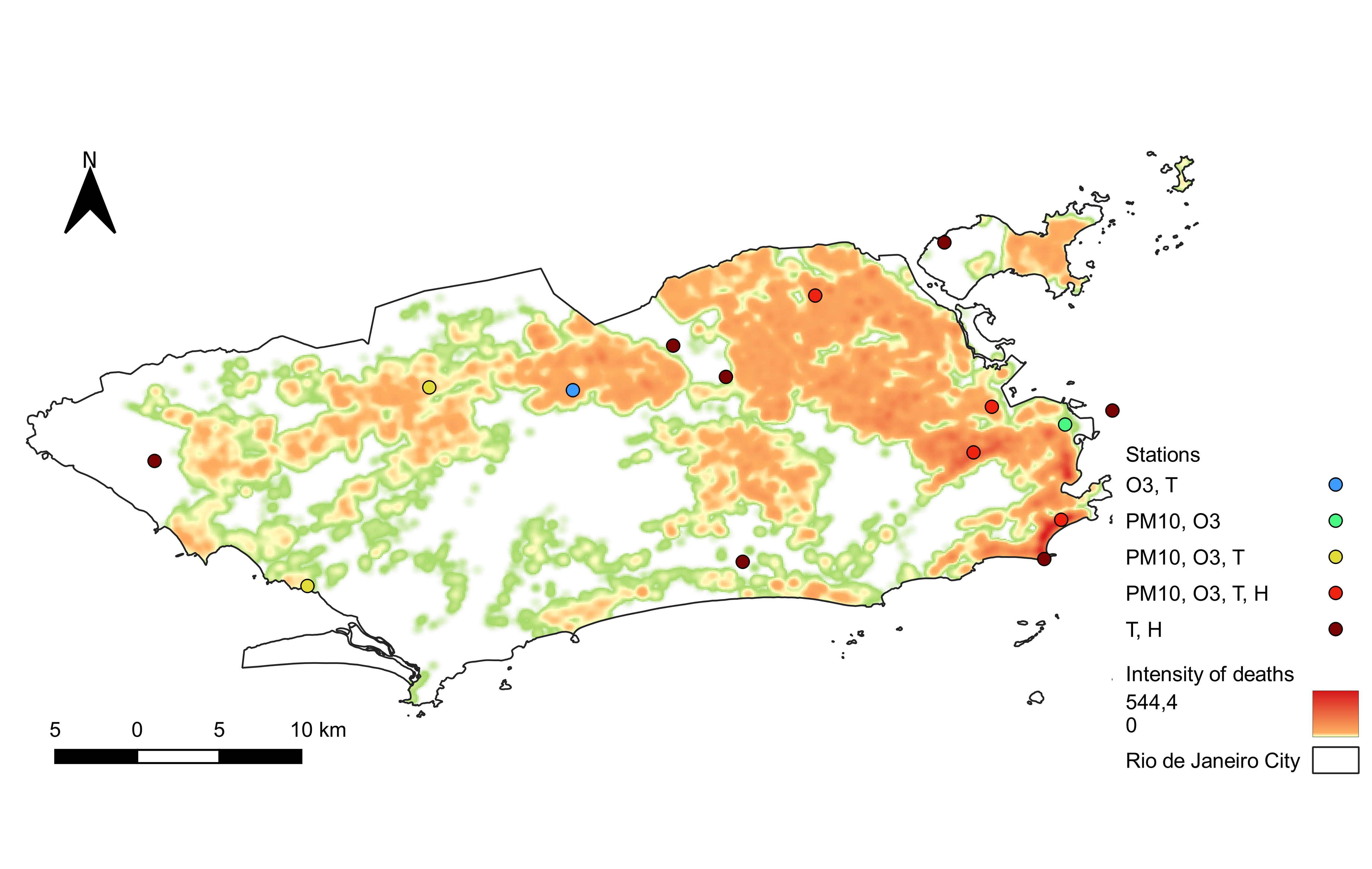

Supplement: S1 Fig — The hottest spots (in red) indicate a high density of deaths within a 500-meter buffer zone. (TIF) [file pone.0281499.s001.tif]
