# Supplementary material for: Short-term association between ambient air pollution and cardio-respiratory mortality in Rio de Janeiro, Brazil
Source: PLoS One. 2023 Feb 16;18(2):e0281499. doi: 10.1371/journal.pone.0281499 (PMC9934392; doi:10.1371/journal.pone.0281499)
Supplement: S1 Table — Stations with more than 25% missing values in a year and/or > 15% missing data over the study period were excluded. (PDF) [file pone.0281499.s002.pdf]

S1 Table. Percentage of imputed missing data.

Station ID	Source	Missing imputed values %			
		PM_{10}	O_3	Temperature	Relative humidity
AV	SMAC	3.4	1.9	1.2	1.2
BG	SMAC	excluded	3.0	excluded	2.6
CA	SMAC	7.0	3.7	excluded	excluded
CG	SMAC	8.9	3.9	1.5	excluded
IR	SMAC	5.9	4.8	1.3	1.9
PG	SMAC	8.2	3.9	2.1	excluded
SC	SMAC	6.9	3.4	1.4	2.1
SP	SMAC	6.0	4.4	2.3	2.0
83054	DCEA	no data	no data	9.1	9.1
83115	DCEA	no data	no data	8.9	8.9
83746	DCEA	no data	no data	1.7	1.7
83748	DCEA	no data	no data	11.4	10.8
83755	DCEA	no data	no data	6.0	6.1
83743	DCEA	no data	no data	excluded	excluded
A621	INMET	no data	no data	1.6	1.7
A625	INMET	no data	no data	0	0

Stations with more than 25% missing values in a year and/or > 15% missing data over the study period were excluded.
