# Supplementary material for: Short-term association between ambient air pollution and cardio-respiratory mortality in Rio de Janeiro, Brazil
Source: PLoS One. 2023 Feb 16;18(2):e0281499. doi: 10.1371/journal.pone.0281499 (PMC9934392; doi:10.1371/journal.pone.0281499)
Supplement: S2 Table — (PDF) [file pone.0281499.s003.pdf]

S2 Table. Root mean square error (RMSE) and mean absolute error (MAE) values of cross-validation for the different sets of inverse distance weighting parameters.

	PM_{10}		O_3		Temperature		Relative humidity	
**Search distance**	RMSE	MAE	RMSE	MAE	RMSE	MAE	RMSE	MAE
5km	0.216	0.194	0.357	0.304	-	-	-	-
10km	0.409	0.322	0.448	0.359	2.027	1.712	6.120	5.200
15km	0.384	0.299	0.447	0.381	1.854	1.560	6.024	5.026
20km	0.372	0.289	0.446	0.359	1.872	1.552	5.826	5.826
>20km	0.355	0.270	0.408	0.328	1.720	1.412	5.811	4.801
