# Supplementary material for: Short-term association between ambient air pollution and cardio-respiratory mortality in Rio de Janeiro, Brazil
Source: PLoS One. 2023 Feb 16;18(2):e0281499. doi: 10.1371/journal.pone.0281499 (PMC9934392; doi:10.1371/journal.pone.0281499)
Supplement: S3 Table — (PDF) [file pone.0281499.s004.pdf]

S3 Table. Descriptive statistics of the study sample and excluded participants.

	Study Sample<br>% (n)	Excluded deaths<br>% (n)
**Total**	112,869	19,994
**Cardiovascular causes**	76,798	14,099
Ischemic heart diseases (I20-25)	25,005 (33)	4,059 (29)
Stroke (I60-I69)	18,438 (24)	3,680 (26)
Heart failure (I50)	4,582 (6)	902 (6)
**Respiratory causes**	36,071	5,895
COPD (J44)	6,146 (17)	896 (15)
Asthma (J45)	400 (1)	67 (1)
**Age group**		
Age < 64 years	27,025 (24)	7,230 (36)
Age ≥ 65 years	85,798 (76)	12,507 (63)
**Gender**		
Female	59,923 (53)	9,975 (50)
Male	52,943 (47)	10,017 (49)
**Color/race**		
Non-white	41,458 (37)	10,821 (54)
White	70,328 (62)	8,920 (45)
**Educational attainment**		
Elementary or none (≤ 7 years)	69,103 (61)	14,783 (74)
High school or above	37,520 (33)	3,644 (18)
**Death – time of year**		
Warm season	35,812 (32)	6,458 (32)
Cold season	59,751 (53)	10,390 (52)
