# Supplementary material for: Short-term association between ambient air pollution and cardio-respiratory mortality in Rio de Janeiro, Brazil
Source: PLoS One. 2023 Feb 16;18(2):e0281499. doi: 10.1371/journal.pone.0281499 (PMC9934392; doi:10.1371/journal.pone.0281499)
Supplement: S4 Table — OR: odds ratio; 95% CI: 95% confidence interval; Models were performed using case-crossover design and adjusted for temperature and relative humidity. (PDF) [file pone.0281499.s005.pdf]

S4 Table. Lag-specific association of short-term exposure to PM 10 and O 3 , and cardiovascular and respiratory mortality, Rio de Janeiro (Brazil), (2012-2017).

**Pollutant**	**Respiratory disease mortality** OR (95% CI)	**Cardiovascular disease mortality** OR (95% CI)
**PM_{10}**		
Lag 0	1.002 (0.992 – 1.012)	0.997 (0.990 – 1.004)
Lag 1	1.000 (0.993 – 1.007)	1.002 (0.997 – 1.006)
Lag 2	1.001 (0.994 – 1.008)	1.002 (0.998 – 1.007)
Lag 3	1.006 (0.995 – 1.017)	1.000 (0.993 – 1.008)
**O_3**		
Lag 0	0.996 (0.989 – 1.003)	1.000 (0.995 – 1.005)
Lag 1	0.997 (0.992 – 1.002)	1.001 (0.997 – 1.004)
Lag 2	0.997 (0.992 – 1.001)	1.001 (0.998 – 1.005)
Lag 3	0.996 (0.989 – 1.003)	1.003 (0.998 – 1.008)

OR: odds ratio; 95% CI: 95% confidence interval; Models were performed using case-crossover design and adjusted for temperature and relative humidity.
