# Supplementary material for: Short-term association between ambient air pollution and cardio-respiratory mortality in Rio de Janeiro, Brazil
Source: PLoS One. 2023 Feb 16;18(2):e0281499. doi: 10.1371/journal.pone.0281499 (PMC9934392; doi:10.1371/journal.pone.0281499)
Supplement: S5 Table — OR: Odds ratio for a 10 μg/m3 increase in each pollutant exposure; 95% CI: 95% confidence interval. ** Risk Ratio and 95% CI estimated using a time series design and Poisson regression models adjusted for mean daily temperature, mean daily absolute humidity, seasonality, and day of the week. (PDF) [file pone.0281499.s006.pdf]

S5 Table. Sensitivity analyses of the short-term mortality effects of PM10 and O3.

Models	Respiratory disease mortality OR (95% CI)		Cardiovascular disease mortality OR (95% CI)	
	PM_{10}	O_3	PM_{10}	O_3
Air pollutants with 5-day lag	1.014 (0.998 – 1.029)	0.989 (0.977 – 1.001)	1.000 (0.990 – 1.011)	1.005 (0.997 – 1.014)
Air pollutants with 10-day lag	1.017 (0.997 – 1.037)	0.986 (0.970 – 1.002)	1.004 (0.991 – 1.018)	1.008 (0.997 – 1.019)
Temperature with 7-day lag	1.009 (0.995 – 1.023)	0.989 (0.978 – 0.999)	1.001 (0.991 – 1.010)	1.005 (0.998 – 1.012)
Temperature with 21-day lag	1.004 (0.990 – 1.018)	0.990 (0.977 – 1.002)	0.998 (0.988 – 1.007)	1.005 (0.998 – 1.013)
Temperature with knots at the 10th, 75th, and 90th percentiles	1.008 (0.994– 1.022)	0.986 (0.976 – 0.996)	1.002 (0.992 – 1.011)	1.005 (0.998 – 1.012)
Temperature b-spline adjusted	1.008 (0.994– 1.022)	0.985 (0.975 – 0.996)	1.001 (0.991 – 1.010)	1.005 (0.998 – 1.012)
No adjustment for humidity	1.009 (0.996 – 1.023)	0.987 (0.977 – 0.997)	1.003 (0.994 – 1.013)	1.006 (0.999 – 1.013)
Adjusted for relative humidity	1.010 (0.996– 1.024)	0.990 (0.978 – 1.002)	1.002 (0.993 – 1.012)	1.005 (0.998 – 1.012)
Only deaths located within 10km of a monitoring station	1.005 (0.991 – 1.018)	0.988 (0.978 – 0.998)	1.000 (0.990 – 1.009)	1.004 (0.998 – 1.011)
Only deaths located within 5km of a monitoring station	1.007 (0.988 – 1.026)	0.984 (0.970 – 0.999)	0.985 (0.973 – 0.998)	1.005 (0.997 – 1.013)
Poisson times series**	1.040 (1.030 – 1.050)	0.995 (0.986 – 1.004)	1.028 (1.021 – 1.035)	1.012 (1.006 – 1.018)

OR: odds ratio for a 10 μg/m 3 increase in each pollutant exposure; 95% CI: 95% confidence interval. ** Risk Ratio and 95% CI estimated using a time series design and Poisson regression models adjusted for mean daily temperature, mean daily absolute humidity, seasonality, and day of the week
