# Supplementary material for: Short-term association between ambient air pollution and cardio-respiratory mortality in Rio de Janeiro, Brazil
Source: PLoS One. 2023 Feb 16;18(2):e0281499. doi: 10.1371/journal.pone.0281499 (PMC9934392; doi:10.1371/journal.pone.0281499)
Supplement: S6 Table — * Differences between daily concentrations of each pollutant on event days (day of death) and average concentrations over the control period. **Difference between pollutant concentrations in event and control days using all the mortality data (geocoded and non-geocoded) and daily average exposure data (all stations without imputation and IDW interpolation). PM10: Particulate matter ≤10 μm; O3: Ozone; SD: Standard deviation; IQR: Interquartile range. (PDF) [file pone.0281499.s007.pdf]

S6 Table. Pollutant concentration differences between event days and average concentrations over the control period in Rio de Janeiro, Brazil (2012–2017).

**Concentration difference between event and control days***	Mean	SD	Median	IQR	Minimum	Maximum
Respiratory deaths						
PM_{10} (µg/m^3)	0.28	16.38	-1.02	20.03	- 61.41	118.27
O_3 (µg/m^3)	0.20	23.56	- 0.97	25.98	- 30.93	185.59
CVD deaths						
PM_{10} (µg/m^3)	0.15	16.18	-1.13	19.76	-61.93	117.91
O_3 (µg/m^3)	0.34	24.13	-1.03	26.99	- 35.99	184.82
CVD and respiratory deaths						
PM_{10} (µg/m^3)	0.19	16.24	-1.09	19.86	-61.93	118.27
O_3 (µg/m^3)	0.29	23.95	-1.01	26.66	-135.99	185.59
All deaths**						
PM_{10} (µg/m^3)	0.20	15.13	-0.89	18.83	- 46.42	71.23
O_3 (µg/m^3)	0.35	19.74	-0.51	24.32	-67.80	75.06

* Differences between daily concentrations of each pollutant on event days (day of death) and average concentrations over the control period. **Difference between pollutant concentrations in event and control days using all the mortality data (geocoded and non-geocoded) and daily average exposure data (all stations without imputation and IDW interpolation). PM10 : particulate matter ≤10 µm; O3 : ozone; SD: standard deviation; IQR: interquartile range
