# Supplementary material for: Short-term association between ambient air pollution and cardio-respiratory mortality in Rio de Janeiro, Brazil
Source: PLoS One. 2023 Feb 16;18(2):e0281499. doi: 10.1371/journal.pone.0281499 (PMC9934392; doi:10.1371/journal.pone.0281499)
Supplement: S1 Appendix — (PDF) [file pone.0281499.s008.pdf]

S1 Appendix. Modeling choices

First, to select the lag period, we examined the relationship between pollutant exposures and mortality outcomes using a maximum lag of 15 days. For respiratory mortality, we found higher associations at lag 0 (and 7) for both pollutants, with the OR decreasing at lag 3 and after lag 10. In relation to cardiovascular diseases, estimates for both pollutants attenuated after lag 2. We therefore used a maximum of 3 lags for the main analyses and presented the results for the different lag periods (lag 0-5, lag 0-10 days) as sensitivity analyses.

Potential confounders were selected using causal directed acyclic graphs – DAG (Greenland et al., 1999). The DAG in the figure below shows our main hypotheses underlying the relationship between air pollution (PM_{10} and O_3) exposure and mortality due to cardio-respiratory diseases. The environmental concentrations of PM_{10} and O_3 - estimated at the residential address -were used as a proxy for individual exposure to air pollution. We assumed that exposure measurement errors were due to individual mobility patterns and other unobserved factors, such as proximity to emission sources, wind speed/direction, solar radiation, etc. (represented by U). Time-varying factors, such as daily temperature and daily humidity, were considered potential confounders, since they can directly or indirectly influence exposure to air pollutants and are associated with an increase in cardio-respiratory mortality. For simplicity, we represent the time-varying factors at only one moment (t) and assume that the causal structure does not change over time. Although some meteorological factors may have long-lagged effects on mortality (e.g., 21 days for temperature), we considered their direct effects on exposure to be short-term. However, in the sensitivity analyses, we tested different lag periods for temperature.

Assuming that our causal DAG is correct, the minimal sufficient adjustment set to estimate the total effect (Textor et al., 2012) of air pollution exposure (PM_{10} and O_3) on cardiovascular and respiratory mortality therefore included the following variables: daily absolute humidity, daily temperature, and subset C, which represents the time-invariant confounders (gender, age etc.) assumed to be adjusted by design.

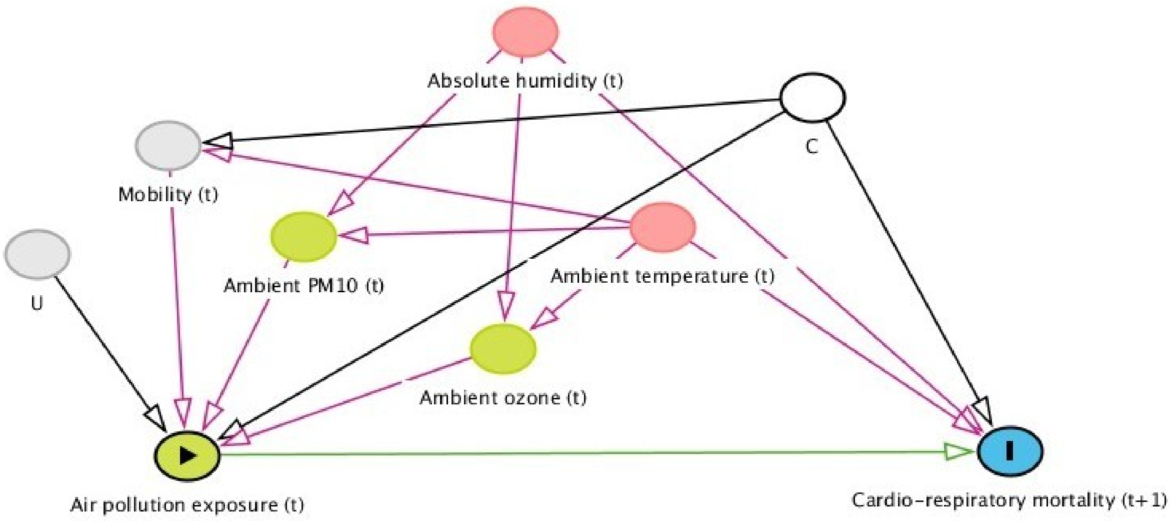

Causal directed acyclic graph depicting the relationships between exposure to ambient air pollution, and cardiovascular and respiratory mortality. The yellow and blue nodes represent the exposure and outcome of interest, respectively. Gray nodes represent unmeasured factors, while the white node C represents individual factors that are adjusted by design.

References

Greenland S, Pearl J, Robins JM. Causal diagrams for epidemiologic research. Epidemiology. 1999 Jan 1:37-48.

Textor J, Hardt J, Knüppel S. DAGitty: a graphical tool for analyzing causal diagrams. Epidemiology. 2011 Sep 1;22(5):745.
