# Supplementary material for: Short-term association between ambient air pollution and cardio-respiratory mortality in Rio de Janeiro, Brazil
Source: PLoS One. 2023 Feb 16;18(2):e0281499. doi: 10.1371/journal.pone.0281499 (PMC9934392; doi:10.1371/journal.pone.0281499)
Supplement: Attachment — Submitted filename: PONE-D-22-17313_reviewer.pdf [file pone.0281499.s009.pdf]

PLOS ONE

Short-term association between ambient air pollution and cardio-respiratory mortality in Rio de Janeiro, Brazil.

--Manuscript Draft--

**Manuscript Number:**	PONE-D-22-17313
**Article Type:**	Research Article
**Full Title:**	Short-term association between ambient air pollution and cardio-respiratory mortality in Rio de Janeiro, Brazil.
**Short Title:**	Air pollution and cardio-respiratory mortality in Rio de Janeiro (Brazil).
**Corresponding Author:**	Taisa Rodrigues Cortes, Ph.D<br>Rio de Janeiro State University: Universidade do Estado do Rio de Janeiro<br>Rio de Janeiro, RJ BRAZIL
**Keywords:**	Air pollution; Mortality; Cardiovascular diseases; Respiratory diseases; Case-crossover studies.
**Abstract:**	Background. Several epidemiological studies have reported associations between ambient air pollution and mortality. However, relatively few studies have investigated this relationship in Brazil using individual-level data. Objectives To estimate the short-term association between exposure to particulate matter <10 µm (PM10) and ozone (O3), and cardiovascular and respiratory mortality in Rio de Janeiro, Brazil, between 2012 and 2017. Methods: We used a time-stratified case-crossover study design with individual-level mortality data. Our sample included 76,798 deaths from cardiovascular diseases and 36,071 deaths from respiratory diseases. Individual exposure to air pollutants was estimated by the inverse distance weighting method using data from seven monitoring stations for PM10 (24-hour mean) and eight stations for O3 (8-hour max). We estimated the mortality effects of PM10 and O3 over a 3-day lag using conditional logistic regression models combined with distributed lag non - linear models. The models were adjusted for daily mean temperature and daily mean absolute humidity. Effect estimates we re presented as odds ratios (OR) with their 95% confidence interval (CI) associated with a 10 µg/m3 increase in each pollutant exposure. Results: No consistent associations were observed for both pollutant and mortality outcome. The cumulative OR of PM10 exposure was 1.01 (95% CI 0.99 – 1.02) for respiratory mortality and 1.00 (95% CI 0.99 – 1.01) for cardiovascular mortality. For O3 exposure, we also found no evidence of increased mortality for cardiovascular (OR 1.01, 95% CI 1.00 – 1.01) or respiratory diseases (OR 0.99, 95% CI 0.98 – 1.00). Our findings were similar across age and gender subgroups, and different model specifications. Conclusion: We found no consistent associations between the PM10 and O3 concentrations observed in our study and cardio-respiratory mortality. Future studies need to explore more refined exposure assessment methods to improve health risk estimates and the planning and evaluation of public health and environmental policies.
**Order of Authors:**	Taisa Rodrigues Cortes, Ph.D<br>Ismael Henrique Silveira, Ph.D<br>Beatriz Fátima Alves Oliveira, Ph.D<br>Washington Leite Junger, Ph.D
**Additional Information:**	
**Question**	**Response**
**Financial Disclosure**<br><br>Enter a financial disclosure statement that describes the sources of funding for the work included in this submission. Review the submission guidelines for detailed	This research was funded by the Coordination for the Improvement of Higher Education Personnel – CAPES (https://www.gov.br/capes/) (finance code 001), Foundation for Research Support of the State of Rio de Janeiro - FAPERJ (https://www.faperj.br/)(grant number E-26/202.756/2018), National Council of Technological and Scientific Development – CNPq (https://www.gov.br/cnpq/)(grant numbers 307495/2018-3 and 406292/2018-3) and the Wellcome Trust

Powered by Editorial Manager® and ProduXion Manager® from Aries Systems Corporation

requirements. View published research articles from *PLOS ONE* for specific examples.<br><br>This statement is required for submission and **will appear in the published article** if the submission is accepted. Please make sure it is accurate.<br><br>**Unfunded studies**<br>Enter: *The author(s) received no specific funding for this work.*<br><br>**Funded studies**<br>Enter a statement with the following details:<br>• Initials of the authors who received each award<br>• Grant numbers awarded to each author<br>• The full name of each funder<br>• URL of each funder website<br>• Did the sponsors or funders play any role in the study design, data collection and analysis, decision to publish, or preparation of the manuscript?<br>• **NO** - Include this sentence at the end of your statement: *The funders had no role in study design, data collection and analysis, decision to publish, or preparation of the manuscript.*<br>• **YES** - Specify the role(s) played.<br><br>* typeset	(https://wellcome.org/) (grant number 216087/Z/19/Z).
**Competing Interests**<br><br>Use the instructions below to enter a competing interest statement for this submission. On behalf of all authors, disclose any competing interests that could be perceived to bias this work—acknowledging all financial support and any other relevant financial or non-financial competing interests.<br><br>This statement is required for submission and **will appear in the published article** if the submission is accepted. Please make sure it is accurate and that any funding sources listed in your Funding Information later in the submission form are also declared in your Financial Disclosure	The authors have declared that no competing interests exist.

Powered by Editorial Manager® and ProduXion Manager® from Aries Systems Corporation

statement.<br><br>View published research articles from *PLOS ONE* for specific examples.<br><br>**NO authors have competing interests**<br><br>Enter: *The authors have declared that no competing interests exist.*<br><br>**Authors with competing interests**<br><br>Enter competing interest details beginning with this statement:<br><br>*I have read the journal's policy and the authors of this manuscript have the following competing interests: [insert competing interests here]*<br><br>* typeset	
**Ethics Statement**<br><br>Enter an ethics statement for this submission. This statement is required if the study involved:<br><br>• Human participants<br>• Human specimens or tissue<br>• Vertebrate animals or cephalopods<br>• Vertebrate embryos or tissues<br>• Field research<br><br>Write "N/A" if the submission does not require an ethics statement.<br><br>General guidance is provided below. Consult the submission guidelines for detailed instructions. **Make sure that all information entered here is included in the Methods section of the manuscript.**	Ethical approval for this study was obtained from the Research Ethics Committees of the Municipal Health Department of Rio de Janeiro and the State University of Rio de Janeiro. The mortality data were anonymized, and the authorization to carry out the research without the informed consent of subjects was given by the ethics committees.

**Format for specific study types**<br><br>**Human Subject Research (involving human participants and/or tissue)**<br>• Give the name of the institutional review board or ethics committee that approved the study<br>• Include the approval number and/or a statement indicating approval of this research<br>• Indicate the form of consent obtained (written/oral) or the reason that consent was not obtained (e.g. the data were analyzed anonymously)<br><br>**Animal Research (involving vertebrate animals, embryos or tissues)**<br>• Provide the name of the Institutional Animal Care and Use Committee (IACUC) or other relevant ethics board that reviewed the study protocol, and indicate whether they approved this research or granted a formal waiver of ethical approval<br>• Include an approval number if one was obtained<br>• If the study involved *non-human primates*, add *additional details* about animal welfare and steps taken to ameliorate suffering<br>• If anesthesia, euthanasia, or any kind of animal sacrifice is part of the study, include briefly which substances and/or methods were applied<br><br>**Field Research**<br><br>Include the following details if this study involves the collection of plant, animal, or other materials from a natural setting:<br>• Field permit number<br>• Name of the institution or relevant body that granted permission	
**Data Availability**<br><br>Authors are required to make all data underlying the findings described fully available, without restriction, and from the time of publication. PLOS allows rare exceptions to address legal and ethical concerns. See the PLOS Data Policy and FAQ for detailed information.	No - some restrictions will apply

Powered by Editorial Manager® and ProduXion Manager® from Aries Systems Corporation

A Data Availability Statement describing where the data can be found is required at submission. Your answers to this question constitute the Data Availability Statement and **will be published in the article**, if accepted.<br><br>**Important:** Stating 'data available on request from the author' is not sufficient. If your data are only available upon request, select 'No' for the first question and explain your exceptional situation in the text box.<br><br>Do the authors confirm that all data underlying the findings described in their manuscript are fully available without restriction?	
**Describe where the data may be found in full sentences. If you are copying our sample text, replace any instances of XXX with the appropriate details.**<br><br>• If the data are **held or will be held in a public repository**, include URLs, accession numbers or DOIs. If this information will only be available after acceptance, indicate this by ticking the box below. For example: *All XXX files are available from the XXX database (accession number(s) XXX, XXX.).*<br>• If the data are all contained **within the manuscript and/or Supporting Information files**, enter the following: *All relevant data are within the manuscript and its Supporting Information files.*<br>• If neither of these applies but you are able to provide **details of access elsewhere**, with or without limitations, please do so. For example:<br><br>*Data cannot be shared publicly because of [XXX]. Data are available from the XXX Institutional Data Access / Ethics Committee (contact via XXX) for researchers who meet the criteria for access to confidential data.*<br><br>*The data underlying the results presented in the study are available from (include the name of the third party*	All the files that are not protected under data protection laws, such as the environmental data and R codes, are available at https://github.com/reprotc.

Powered by Editorial Manager® and ProduXion Manager® from Aries Systems Corporation

and contact information or URL).<br>• This text is appropriate if the data are owned by a third party and authors do not have permission to share the data.<br><br>* typeset	
Additional data availability information:	Tick here if the URLs/accession numbers/DOIs will be available only after acceptance of the manuscript for publication so that we can ensure their inclusion before publication.

Powered by Editorial Manager® and ProduXion Manager® from Aries Systems Corporation

Manuscript
Click here to access/download;Manuscript;paper_plosone_pollution_rio.doc

Short-term association between ambient air pollution and cardio-respiratory mortality in Rio de Janeiro, Brazil.

Taísa Rodrigues Cortes[1*], Ismael Henrique Silveira[2], Beatriz Fátima Alves de Oliveira[3], and Washington Leite Junger[1].

1. Institute of Social Medicine, State University of Rio de Janeiro, Rio de Janeiro, Brazil.
2. Institute of Public Health, Federal University of Bahia, Salvador, Brazil.
3. Fiocruz Regional Office in Piauí, National School of Public Health, Oswaldo Cruz Foundation, Teresina, Brazil.

*Corresponding author:
Taísa Rodrigues Cortes
Instituto de Medicina Social.
Universidade do Estado do Rio de Janeiro.
São Francisco Xavier, 524 - 7º andar - Maracanã, Rio de Janeiro - RJ, Brasil. CEP: 20550-90.
E-mail: taisacortes@gmail.com

Abstract

Background. Several epidemiological studies have reported associations between ambient air pollution and mortality. However, relatively few studies have investigated this relationship in Brazil using individual-level data. **Objectives** To estimate the short-term association between exposure to particulate matter <10 μm (PM_{10}) and ozone (O_3), and cardiovascular and respiratory mortality in Rio de Janeiro, Brazil, between 2012 and 2017. **Methods:** We used a time-stratified case-crossover study design with individual-level mortality data. Our sample included 76,798 deaths from cardiovascular diseases and 36,071 deaths from respiratory diseases. Individual exposure to air pollutants was estimated by the inverse distance weighting method using data from seven monitoring stations for PM_{10} (24-hour mean) and eight stations for O_3 (8-hour max). We estimated the mortality effects of PM_{10} and O_3 over a 3-day lag using conditional logistic regression models combined with distributed lag non-linear models. The models were adjusted for daily mean temperature and daily mean absolute humidity. Effect estimates were presented as odds ratios (OR) with their 95% confidence interval (CI) associated with a 10 μg/m3 increase in each pollutant exposure. **Results:** No consistent associations were observed for both pollutant and mortality outcome. The cumulative OR of PM_{10} exposure was 1.01 (95% CI 0.99 – 1.02) for respiratory mortality and 1.00 (95% CI 0.99 – 1.01) for cardiovascular mortality. For O_3 exposure, we also found no evidence of increased mortality for cardiovascular (OR 1.01, 95% CI 1.00 – 1.01) or respiratory diseases (OR 0.99, 95% CI 0.98 – 1.00). Our findings were similar across age and gender subgroups, and different model specifications. **Conclusion:** We found no consistent associations between the PM_{10} and O_3 concentrations observed in our study and cardio-respiratory mortality. Future studies need to explore more refined exposure assessment methods to improve health risk estimates and the planning and evaluation of public health and environmental policies.

1. Introduction

The World Health Organization (WHO) estimated that approximately 3 million deaths could be attributed to ambient air pollution in 2012 [1]. Mortality from respiratory and cardiovascular diseases are among the most commonly investigated health outcomes associated with air pollution [2]. Cardiovascular diseases are the leading cause of non-communicable diseases (NCDs) and accounted for nearly 18 million deaths in 2019 [3]. Chronic respiratory diseases also carry a significant burden of premature mortality and disability, and were the third leading cause of death in 2017 [4]. Non-communicable diseases are an important public health challenge worldwide, and air pollution is now ranked among their major risk factors [5-6].

Ambient air pollution consists of a complex and heterogeneous mixture of hazardous components. The health damage caused by air pollutants depends upon their levels and composition in the environment; the duration, frequency, and timing that individuals are exposed; and individual susceptibility [7]. Pollutant levels and composition are influenced not only by emission intensity and source (such as motor vehicles, industry, and biomass burning) but also by landscape features

and meteorological processes [8-9]. Differential population vulnerabilities to air pollution might also be due to differences in socioeconomic conditions and demographic characteristics [10-11].

Since many of the factors affecting individual exposure and vulnerabilities can vary significantly between different populations and geographic areas, investigations into the health effects of air pollution are particularly relevant at regional and local level. In this study, we focus on two major pollutants, ambient particulate matter with an aerodynamic diameter ≤10 μm (PM_{10}) and ground-level ozone (O_3). Although several epidemiological studies have reported associations between ambient air pollution and mortality [2,12-14], relatively few studies in Brazil have investigated this relationship using individual-level data. In this study, we used a case-crossover design with individual-level mortality data to estimate the short-term association between exposure to PM_{10} and O_3, and cardiovascular and respiratory disease mortality in Rio de Janeiro, Brazil. We also investigate effect modification by age and gender.

2. Methods

2.1 Mortality data

We carried out a time-stratified case-crossover study to investigate the association between short-term exposure to PM_{10} and O_3, and cardiovascular and respiratory disease mortality in Rio de Janeiro (Brazil). Mortality data were obtained from the Municipal Health Department of Rio de Janeiro. We selected all deaths from cardiovascular (International Classification of Diseases, 10th Revision – ICD-10, codes I00-I99) and respiratory diseases (ICD-10, codes J00-J99) that occurred in residents of the municipality of Rio de Janeiro between 2012 and 2017. Residential addresses were geocoded and validated using the method described in a previous study [15]. In brief, out of the total deaths (n=132,863), approximately 86% (n=113,876) were geocoded to street level, with a sensitivity and specificity of 87% and 98%, respectively.

Ethical approval for this study was obtained from the Research Ethics Committees of the Municipal Health Department of Rio de Janeiro and the State University of Rio de Janeiro. The mortality data were anonymized, and the authorization to carry out the research without the informed consent of subjects was given by the ethics committees.

2.2 Environmental data

Hourly air pollution and meteorological data were obtained from the monitoring stations maintained by Rio de Janeiro's Municipal Secretariat for the Environment. Additional

meteorological data were obtained from the Brazilian Institute of Meteorology and from airport weather stations maintained by Brazil's National Institute for Space Research.

We calculated daily concentrations of PM_{10} (µg/m^3), maximum daily 8-hour O_3 (µg/m^3), mean daily temperature (Cº), and mean relative humidity (%). For the computation of pollutant daily averages, availability of greater than 75% of observations was required. We only included monitoring station data in cases where less than 15% of the study period data was missing (7 stations for PM_{10}, 8 for O_3, 13 for temperature, and 12 for relative humidity). Absolute humidity was estimated using relative humidity and temperature data, as previously described [16].

The imputation of missing data was performed using a method based on the expectation-maximization algorithm implemented in the mtsdi package [17]. Missing values were estimated using the covariance matrix of the stations' data. The series temporal component (trend and seasonality) was modeled using natural cubic splines with 5 degrees of freedom per year. The amount of missing data imputed in each time series is presented in S1 Table.

2.3 Exposure Assessment

Individual-level exposures to air pollutants and meteorological variables were estimated using the inverse distance weighting (IDW) method. In this method, the values at unmeasured locations are estimated by averaging the weighted sum of values from the nearest neighbors (monitoring stations) within a search radius. The weights assigned to each neighboring value are expressed as a function of the inverse distance (between the measured and unmeasured locations) raised to a non-negative power [18].

Leave-one-out cross-validation was carried out to determine the optimal power value, the number of nearest neighbors, and the search radius. For each day, we selected the set of parameters that minimized the prediction root mean square error (RMSE) [19]. The power of distance was evaluated using five values from 1 to 3, and the number of neighbors ranged from 1 to the maximum number of stations (7, 8, 12, or 13). Restricting the search radius to 10km, 15km, or 20km did not improve interpolation results. Although we observed better results for air pollutants using the 5km radius, these data were only used in the sensitivity analysis due to the high number of grids with missing data. The root mean square error (RMSE) and mean absolute error (MAE) values of cross-validation for the different sets of parameters are summarized in S2 Table.

Daily weighted averages of PM_{10}, O_3, and meteorological variables were interpolated into a one square kilometer grid [20]. Individual exposure estimates were calculated through the average of the interpolated values within a 1-kilometer buffer of each residential address.

2.4 Statistical analysis

The short-term mortality effects of PM_{10} and O_3 were estimated using conditional logistic regression models combined with distributed lag non-linear models [21]. In line with most previous studies [12], we assumed a linear relationship between air pollution concentration and mortality outcomes. We considered the effects of each pollutant exposure on the day of death (lag 0) and exposures from 1 to 3 days preceding death (lag 1 to lag 3). The lag–response dimension was defined using a natural cubic spline function with three degrees of freedom.

We used a time-stratified case-crossover study with control days selected on the same day of the week and in the same month and year of death [22]. Time-invariant confounders were adjusted by design, as a consequence of self-matching. Time-variant factors, such as daily temperature and daily humidity, were considered potential confounders (as depicted in the causal directed acyclic graph (DAG) in S1 File). We used a cross-basis function to account for the potential nonlinear confounding effects of temperature [21]. The exposure-response dimension of the cross-basis function for temperature was defined using a natural cubic spline with two internal knots (at the 50th and 75th percentiles), while the lag dimension was modeled with a natural cubic spline with two degrees of freedom and a maximum lag of up to 3 days. Daily absolute humidity (lag 0-3 average) was adjusted using a natural cubic spline with one internal knot. We controlled for absolute humidity rather than relative humidity, since the former is a direct measure of water vapor in the air, regardless of air temperature, thus potentially minimizing over-fitting and multicollinearity [23-24]. The number of knots and their respective locations were selected based on the Akaike Information Criteria [25]. More details about our modeling choices are provided in S1 appendix.

Estimates of lag-specific (total) and cumulative effects are presented as odds ratios (OR), and their 95% confidence interval (CI), associated with a 10 μg/m3 increase in each pollutant exposure. We performed stratified analysis by gender (female, male) and age group. In the age-stratified analysis, the deaths were categorized into two age groups (<64 years and ≥ 65 years).

We carried out several sensitivity analyses to examine the robustness of our model estimates. We investigated lag periods of 5 and 10 days for PM_{10} and O_3, and 7 and 21 days for temperature[illegible]e also checked that the results were robust to different adjustments for humidity (a model with relative humidity and one without any humidity adjustment) and for temperature. In addition, since pollutant concentrations may have high intra-urban spatial variation, we restricted our analysis to deaths whose geocoded address was located within 10km and 5km of a monitoring

station. Finally, we estimated the cumulative effect over a 3-day lag using a time series design and Poisson regression models. The models were adjusted for daily temperature, absolute humidity, long-term trends (natural cubic spline of time with 5 degrees of freedom per year), and day of the week (as an indicator variable).

All analyses were conducted using R 4.1.0. We used different R packages, including the packages survival [26] and dlnm [21] to fit the main models, and the package gstat for interpolation [27]. All the files that are not protected under data protection laws, such as the environmental data and R codes, are available at https://github.com/.

3. Results

During the study period (2012-2017), 90,897 deaths from cardiovascular disease and 41,966 deaths from respiratory diseases were recorded. We excluded those deaths with non-geocoded addresses and lagged exposure prior to 2012. A total of 76,798 deaths due to cardiovascular disease and 36,071 deaths due to respiratory disease were thus included in our analysis. S3 Table presents descriptive statistics for the study sample and the excluded participants.

Summary statistics for air pollutants and meteorological variables are provided in Table 1. The means (and standard deviations) of air pollutants were 37.8 (13.6) μg/m^3 for PM_{10} and 54.3 (19.7) μg/m^3 for O_3.

Table 1. Summary statistics of pollutant concentrations and meteorological variables in Rio de Janeiro, Brazil (2012–2017)

	Mean	SD	Median	IQR	Minimum	Maximum
PM_{10} (μ/m^3)	37.76	13.57	35.48	16.95	11.46	107.76
O_3 (μ/[illegible]^3)	54.31	19.64	51.97	27.01	11.32	143.97
Daily temperature (ºC)	25.36	3.16	25.15	4.73	15.51	33.97
Daily relative humidity (%)	71.84	8.99	72.23	11.90	44.56	94.72

PM_{10}: particulate matter ≤10 μm; O_3: ozone; SD: standard deviation; IQR: interquartile range

The lag-specific mortality association for a 10 μg/m^3 increase in air pollution exposure is shown in Fig 1 (and S4 Table). For PM_{10}, the results indicate a slight increase in respiratory

mortality at lag 0, lag 2 and lag 3. The PM_{10} effect estimate for cardiovascular mortality was slightly higher at lag 1 and lag 2. As with ozone, we found non-significant inverse associations with respiratory mortality over the 3-day lag, while for cardiovascular mortality most point estimates were close to the null value.

Fig 1. Lag-specific association of short-term exposure to PM10 and O3, and respiratory (A-B) and cardiovascular disease mortality (C-D), Rio de Janeiro (Brazil) (2012-2017). PM10: particulate matter ≤10 μm3; O3: ozone; OR: odds ratio.

No consistent cumulative associations were observed for both pollutants and mortality outcomes. As shown in Table 2, the overall estimate of the cumulative effect over a 3-day lag for a 10 μg/m^3 increase in PM_{10} exposure was 1.01 (95% CI 0.99 – 1.02) for respiratory mortality and 1.00 (95% CI 0.99 – 1.01) for cardiovascular mortality. Neither did we find evidence of increased mortality for cardiovascular (OR 1.01, 95% CI 1.00 – 1.01) or respiratory diseases (OR 0.99, 95% CI 0.98 – 1.00) for O_3.

Table 2. Cumulative associations of short-term exposure to PM_{10} and O_3, and cardiovascular and respiratory mortality, Rio de Janeiro (Brazil), (2012-2017).

Pollutant	Subgroup	Respiratory disease mortality OR (95% CI)	Cardiovascular disease mortality OR (95% CI)
**PM_{10}**	All	1.01 (0.99 – 1.02)	1.00 (0.99 – 1.01)
	Age ≥ 65 years	1.01 (0.99 – 1.02)	1.00 (0.99 – 1.01)
	Age 0-64 years	1.01 (0.98 – 1.05)	1.00 (0.98 – 1.02)
	Female	1.01 (0.99 – 1.03)	1.01 (0.99 – 1.02)
	Male	1.01 (0.99 – 1.03)	1.00 (0.98 – 1.01)
**O_3**	All	0.99 (0.98 – 1.00)	1.01 (1.00 – 1.01)
	Age ≥ 65 years	0.99 (0.98 – 1.00)	1.00 (1.00 – 1.01)
	Age 0-64 years	0.97 (0.95 – 0.99)	1.01 (0.99 – 1.02)
	Female	0.99 (0.97 – 1.00)	1.01 (1.00 – 1.02)
	Male	0.98 (0.97 – 1.00)	1.00 (0.99 – 1.01)

OR: odds ratio for a 10 μg/m3 increase in each pollutant exposure.; 95% CI: 95% confidence interval; PM_{10}: particulate matter ≤10 μm; O_3: ozone. Models were performed using case-crossover design and adjusted for daily mean temperature and absolute humidity.

Table 2 shows the air pollution-mortality associations according to the age and gender subgroups. Although we found no evidence of effect modification, the association between PM_{10} and mortality outcomes was positive (although not significant) for most subgroups.

Sensitivity analyses using different model specifications are presented in S5 Table. We found similar results when we removed subjects whose homes were located 10km or 5km away from the nearest monitoring station. Findings also did not change when using different lag periods for pollutant exposures, and different adjustments for temperature and humidity. Notably, the estimates from the minimally adjusted model (without temperature and humidity variables) for both respiratory and cardiovascular mortality were also consistent with slightly positive (but non-significant) effects (data not shown). However, the analyses using the time series design and Poisson regression models show an increase in respiratory mortality associated with changes in PM_{10} concentration. In addition, we found positive associations between both pollutants (PM_{10} and O_3) and cardiovascular disease (CVD) mortality (S5 Table).

4. Discussion

In this study, we investigated the effects of ambient PM_{10} and O_3 exposure on mortality from respiratory and cardiovascular diseases among residents of Rio de Janeiro, Brazil. We found no consistent association between the pollutant concentrations observed in our study and cardio-respiratory mortality. Moreover, we found no evidence of increased mortality effects from air pollution among the age and gender subgroups.

Our results differ from most of the literature regarding the mortality effects of air pollution [12,13, 28, 29]. In a systematic review and meta-analysis of 196 studies assessing the short-term mortality effects of air pollution on various continents, Orellano et al. [12] found significant positive associations between ambient levels of PM_{10} and cardiovascular and respiratory mortality, while for O_3 the authors only found an association with all-cause mortality. A multi-city study in Latin America also found positive associations between ambient PM_{10} levels and cardio-respiratory mortality in most of the cities, including Rio de Janeiro, Porto Alegre, and São Paulo in Brazil [29]. In Rio de Janeiro, PM_{10} concentrations were associated with a 2.14% (95% CI 1.41 – 2.87) increase in respiratory mortality and 0.51% (95% CI 0.06 – 0.96) increase in cardiovascular mortality, while O_3 was only associated with cardiovascular mortality [29]. In contrast, a case-crossover study carried out in São Paulo found that higher concentrations of O_3 was only associated with respiratory mortality [30].

Although some studies have produced similar results to our own, especially for O_3 [12,29,30], evidence from several epidemiological and toxicological studies (both in humans and animals) supports the relationship between air pollution exposure and cardio-respiratory morbidity and mortality [31]. While each pollutant has its own specific toxic properties, increasing evidence indicates oxidative stress and inflammation as key processes underlying the health effects of air pollution [6,32,33]. Air pollutants such as particulate components (PM) and ozone can cause oxidative stress within the lungs, leading to an inflammatory cascade in tissues which can itself lead to systemic inflammation and cell damage [33]. These responses can impact the lung and other organs, either directly or as a secondary effect [34]. Various acute effects of air pollution on the respiratory system have been described in the literature, including the onset and worsening of asthma, and chronic obstructive pulmonary disease (COPD) admissions and mortality. Other recent findings indicate that short-term exposure to major air pollutants (including $PM_{2,5\text{-}10}$ and ozone) can increase the risk of hospitalization and mortality due to ischemic heart diseases [6], heart failure and ischemic stroke [35], particularly in high-risk populations.

Several factors could account for the heterogeneity of the air pollution-mortality association across studies, including differences in research methods and population characteristics. Nonetheless, we believe that the primary reason for the different findings in our study, compared to others, arises from the characteristics of our air pollution data. The daily variability in pollutant concentrations in this study was very low, such that the concentration difference between event and control days had a mean value close to zero (S6 Table). As discussed by Kunzli and Schindler [36], similar exposure levels between case and control periods can provide low statistical power for the detection of pollutant effects in case-crossover studies. Our sensitivity analysis, using a Poisson time series, supports this hypothesis, since we found consistent positive associations between PM_{10} and respiratory disease mortality, and between both pollutants (PM_{10} and O_3) and CVD mortality (S5 Table). However, compared to case-crossover studies, time series studies are more prone to residual confounding due to seasonality and long-term trends compared to case-crossover studies, and thus potential bias might still exist.

Another study limitation is the use of fixed monitoring stations to estimate air pollution exposure. We attempted to reduce exposure error by capturing more of the spatial variability in air pollutants through an interpolation method (IDW). However, this still represents a coarse exposure approach, since it does not account for spatial heterogeneity due to land use (i.e., roads) and meteorological conditions (i.e., wind, solar radiation). Our methods also did not consider the essential determinants of individual exposure, such as daily mobility and indoor air pollution. This may have led to independent and non-differential measurement errors [37], since the influence of

individual activity patterns and time spent indoors/outdoors may have been partially "adjusted" due to self-matching in the case-crossover design (S1 Appendix).

While the mortality data in Brazil has been assessed as good quality [38], the exclusion of deaths due to missing geocoded addresses (14%) could also have affected our results. Our excluded sample had a higher proportion of people of color (54% vs. 37%) and lower educational attainment (74% vs. 61%), which are usually markers for residing in lower income neighborhoods, those with greater segregation and where the cartographic base may be of lower quality. Although we believe that the selection bias magnitude was not high, its impact depends on whether these locations also have higher levels of air pollution or more significant exposure variability.

In conclusion, we found no consistent associations between the PM_{10} and O_3 concentrations observed in our study and cardio-respiratory mortality. To the best of our knowledge, this is the first study using individual-level data that has investigated the association between PM_{10} and O_3 and cardio-respiratory mortality among residents of all ages in Rio de Janeiro, Brazil. The lack of high spatial and temporal resolution air pollution data remains a significant challenge for air pollution research. Future investigations using other study designs should benefit from more refined exposure assessments [39], including satellite data, air pollution modeling, and individual mobility patterns. Enhancing exposure assessment is essential for improving health risk estimates. Adequate monitoring of population exposure to air pollutants is also crucial for planning and evaluating public health and sustainable development policies.

References

1 World Health Organization. Ambient air pollution: A global assessment of exposure and burden of disease. 2016. Available at: https://apps.who.int/iris/bitstream/handle/10665/250141/9789241511353-eng.pdf

2 Sun Z, Zhu D. Exposure to outdoor air pollution and its human health outcomes: A scoping review. PLoS ONE. 2019 May 16;14(5):e0216550. doi: 10.1371/journal.pone.0216550

3 Roth GA, Mensah GA, Johnson CO, Addolorato G, Ammirati E, Baddour LM, et al. Global Burden of Cardiovascular Diseases and Risk Factors, 1990–2019. Journal of the American College of Cardiology. 2020 Dec;76(25):2982–3021. doi: 10.1016/j.jacc.2020.11.010

4 Soriano JB, Kendrick PJ, Paulson KR, Gupta V, Abrams EM, Adedoyin RA, et al. Prevalence and attributable health burden of chronic respiratory diseases, 1990–2017: a systematic analysis for the Global Burden of Disease Study 2017. The Lancet Respiratory Medicine. 2020 Jun;8(6):585–96. doi: 10.1016/S2213-2600(20)30105-3

5 Murray CJL, Aravkin AY, Zheng P, Abbafati C, Abbas KM, Abbasi-Kangevari M, et al. Global burden of 87 risk factors in 204 countries and territories, 1990–2019: a systematic analysis for the Global Burden of Disease Study 2019. The Lancet. 2020 Oct;396(10258):1223–49. doi: 10.1016/S0140-6736(20)30752-2

6 Brook RD, Rajagopalan S, Pope CA, Brook JR, Bhatnagar A, Diez-Roux AV, et al. Particulate Matter Air Pollution and Cardiovascular Disease: An Update to the Scientific Statement From the American Heart Association. Circulation. 2010 Jun;121(21):2331–78. doi: 10.1161/CIR.0b013e3181dbece1

7 Manisalidis I, Stavropoulou E, Stavropoulos A, Bezirtzoglou E. Environmental and Health Impacts of Air Pollution: A Review. Front Public Health. 2020 Feb 20;8:14. doi: 10.3389/fpubh.2020.00014

8 Pérez IA, García MÁ, Sánchez ML, Pardo N, Fernández-Duque B. Key Points in Air Pollution Meteorology. IJERPH. 2020 Nov 11;17(22):8349. doi: 10.3390/ijerph17228349

9 Baklanov A, Molina LT, Gauss M. Megacities, air quality and climate. Atmospheric Environment. 2016 Feb;126:235–49. doi: 10.1016/j.atmosenv.2015.11.059

10 Bell ML, Zanobetti A, Dominici F. Evidence on Vulnerability and Susceptibility to Health Risks Associated With Short-Term Exposure to Particulate Matter: A Systematic Review and Meta-Analysis. American Journal of Epidemiology. 2013 Sep 15;178(6):865–76. doi: 10.1093/aje/kwt090

11 Bell ML, Zanobetti A, Dominici F. Who is More Affected by Ozone Pollution? A Systematic Review and Meta-Analysis. American Journal of Epidemiology. 2014 Jul 1;180(1):15–28. doi: 10.1093/aje/kwu115

12 Orellano P, Reynoso J, Quaranta N, Bardach A, Ciapponi A. Short-term exposure to particulate matter (PM10 and PM2.5), nitrogen dioxide (NO2), and ozone (O3) and all-cause and cause-specific mortality: Systematic review and meta-analysis. Environment International. 2020 Sep;142:105876. doi: 10.1016/j.envint.2020.105876

13 Newell K, Kartsonaki C, Lam KBH, Kurmi OP. Cardiorespiratory health effects of particulate ambient air pollution exposure in low-income and middle-income countries: a systematic review and meta-analysis. The Lancet Planetary Health. 2017 Dec;1(9):e368–80. doi: 10.1016/S2542-5196(17)30166-3

14 Niu Z, Liu F, Yu H, Wu S, Xiang H. Association between exposure to ambient air pollution and hospital admission, incidence, and mortality of stroke: an updated systematic review and meta-analysis of more than 23 million participants. Environ Health Prev Med. 2021 Dec;26(1):15. doi: 10.1186/s12199-021-00937-1

15 Cortes TR, Silveira IH da, Junger WL. Improving geocoding matching rates of structured addresses in Rio de Janeiro, Brazil. Cad Saúde Pública. 2021;37(7):e00039321. doi: 10.1590/0102-311X00039321

16 Silveira IH, Cortes TR, de Oliveira BFA, Junger WL. Projections of excess cardiovascular mortality related to temperature under different climate change scenarios and regionalized climate model simulations in Brazilian cities. Environmental Research. 2021 Jun;197:110995. doi: 10.1016/j.envres.2021.110995

17 Junger WL, Ponce de Leon A. Imputation of missing data in time series for air pollutants. Atmospheric Environment. 2015 Feb;102:96–104. doi: 10.1016/j.atmosenv.2014.11.049

18 Wong DW, Yuan L, Perlin SA. Comparison of spatial interpolation methods for the estimation of air quality data. J Expo Sci Environ Epidemiol. 2004 Sep 1;14(5):404–15. doi: 10.1038/sj.jea.7500338

19 Li Z, Wang P. Intelligent Optimization on Power Values for Inverse Distance Weighting. In: 2013 International Conference on Information Science and Cloud Computing Companion. Guangzhou: IEEE; 2013 p. 370–5. doi: 10.1109/ISCC-C.2013.81

20 Hengl T. Finding the right pixel size. Computers & Geosciences. 2006 Nov;32(9):1283–98. doi: 10.1016/j.cageo.2005.11.008

21 Gasparrini A. Distributed lag linear and non-linear models in R: the package dlnm. Journal of statistical software. 2011;43(8):1

22 Janes H, Sheppard L, Lumley T. Case-Crossover Analyses of Air Pollution Exposure Data: Referent Selection Strategies and Their Implications for Bias. Epidemiology. 2005 Nov;16(6):717–26. doi: 10.1097/01.ede.0000181315.18836.9d

23 Davis RE, McGregor GR, Enfield KB. Humidity: A review and primer on atmospheric moisture and human health. Environmental Research. 2016 Jan;144:106–16. doi: 10.1016/j.envres.2015.10.014

24 Barnett AG, Tong S, Clements ACA. What measure of temperature is the best predictor of mortality? Environmental Research. 2010 Aug;110(6):604–11. doi: 10.1016/j.envres.2010.05.006

25 Craiu RV, Duchesne T, Fortin D. Inference Methods for the Conditional Logistic Regression Model with Longitudinal Data. Biom J. 2008 Feb;50(1):97–109. doi: 10.1002/bimj.200610379

26 Therneau T. A Package for Survival Analysis in R. *R package version* 3.3-1, 2022. https://CRAN.R-project.org/package=survival.

27 Gräler B, Pebesma E, Heuvelink G. Spatio-Temporal Interpolation using gstat. The R Journal. 2016;8(1):204. doi:10.32614/RJ-2016-014
28 Liu C, Chen R, Sera F, Vicedo-Cabrera AM, Guo Y, Tong S, et al. Ambient Particulate Air Pollution and Daily Mortality in 652 Cities. N Engl J Med. 2019 Aug 22;381(8):705–15. doi: 10.1056/NEJMoa1817364
29 Romieu I, Gouveia N, Cifuentes LA, de Leon AP, Junger W, Vera J, et al. Multicity study of air pollution and mortality in Latin America (the ESCALA study). Res Rep Health Eff Inst. 2012 Oct;(171):5–86
30 Bravo MA, Son J, de Freitas CU, Gouveia N, Bell ML. Air pollution and mortality in São Paulo, Brazil: Effects of multiple pollutants and analysis of susceptible populations. J Expo Sci Environ Epidemiol. 2016 Mar;26(2):150–61. doi: 10.1038/jes.2014.90
31 WHO Regional Office for Europe. Review of evidence on health aspects of air pollution – REVIHAAP Project: Technical Report. WHO Regional Office for Europe. 2013.
32 Arias-Pérez RD, Taborda NA, Gómez DM, Narvaez JF, Porras J, Hernandez JC. Inflammatory effects of particulate matter air pollution. Environ Sci Pollut Res. 2020 Dec;27(34):42390–404. doi: 10.1007/s11356-020-10574-w
33 Gangwar RS, Bevan GH, Palanivel R, Das L, Rajagopalan S. Oxidative stress pathways of air pollution mediated toxicity: Recent insights. Redox Biology. 2020 Jul;34:101545. doi: 10.1016/j.redox.2020.101545
34 Schraufnagel DE, Balmes JR, Cowl CT, De Matteis S, Jung SH, Mortimer K, et al. Air Pollution and Noncommunicable Diseases. Chest. 2019 Feb;155(2):417–26. doi: 10.1016/j.chest.2018.10.041
35 Yang WS, Wang X, Deng Q, Fan WY, Wang WY. An evidence-based appraisal of global association between air pollution and risk of stroke. International Journal of Cardiology. 2014 Aug;175(2):307–13. doi: 10.1016/j.ijcard.2014.05.044
36 Kunzli N. A call for reporting the relevant exposure term in air pollution case-crossover studies. Journal of Epidemiology & Community Health. 2005 Jun 1;59(6):527–30. doi: 10.1136/jech.2004.027391
37 van Smeden M, Lash TL, Groenwold RHH. Reflection on modern methods: five myths about measurement error in epidemiological research. International Journal of Epidemiology. 2020 Feb 1;49(1):338–47. doi: 10.1093/ije/dyz251
38 World Health Organization. WHO Methods and Data Sources for Country-Level Causes of Death 2000-2019: WHO; 2020. Available at: https://cdn.who.int/media/docs/default-source/gho-documents/global-health-estimates/ghe2019_cod_methods.pdf (accessed May 2022)
39 Dionisio KL, Baxter LK, Burke J, Özkaynak H. The importance of the exposure metric in air pollution epidemiology studies: When does it matter, and why? Air Qual Atmos Health. 2016 Jul;9(5):495–502. doi: 10.1007/s11869-015-0356-1

Supporting information

Title: Short-term association between ambient air pollution and cardio-respiratory mortality in Rio de Janeiro, Brazil.

S1 Appendix. Modeling choices

S1 Table. Percentage of imputed missing data. Stations with more than 25% missing values in a year and/or > 15% missing data over the study period were excluded.

S2 Table. Root mean square error (RMSE) and mean absolute error (MAE) values of cross-validation for the different sets of inverse distance weighting parameters.

S3 Table. Descriptive statistics of the study sample and excluded participants.

S4 Table. Lag-specific association of short-term exposure to PM_{10} and O_3, and cardiovascular and respiratory mortality, Rio de Janeiro (Brazil), (2012-2017). OR: odds ratio; 95% CI: 95% confidence interval; Models were performed using case-crossover design and adjusted for temperature and relative humidity.

S5 Table. Sensitivity analyses of the short-term mortality effects of PM_{10} and O_3.
OR: odds ratio for a 10 μg/m^3 increase in each pollutant exposure; 95% CI: 95% confidence interval. ** Risk Ratio and 95% CI estimated using a time series design and Poisson regression models adjusted for mean daily temperature, mean daily absolute humidity, seasonality, and day of the week.

S6 Table. Pollutant concentration differences between event days and average concentrations over the control period in Rio de Janeiro, Brazil (2012–2017).

***** Differences between daily concentrations of each pollutant on event days (day of death) and average concentrations over the control period. **Difference between pollutant concentrations in event and control days using all the mortality data (geocoded and non-geocoded) and daily average exposure data (all stations without imputation and IDW interpolation). PM_{10}: particulate matter ≤10 μm; O_3: ozone; SD: standard deviation; IQR: interquartile range

Fig 1

Click here to access/download;Figure;Fig 1

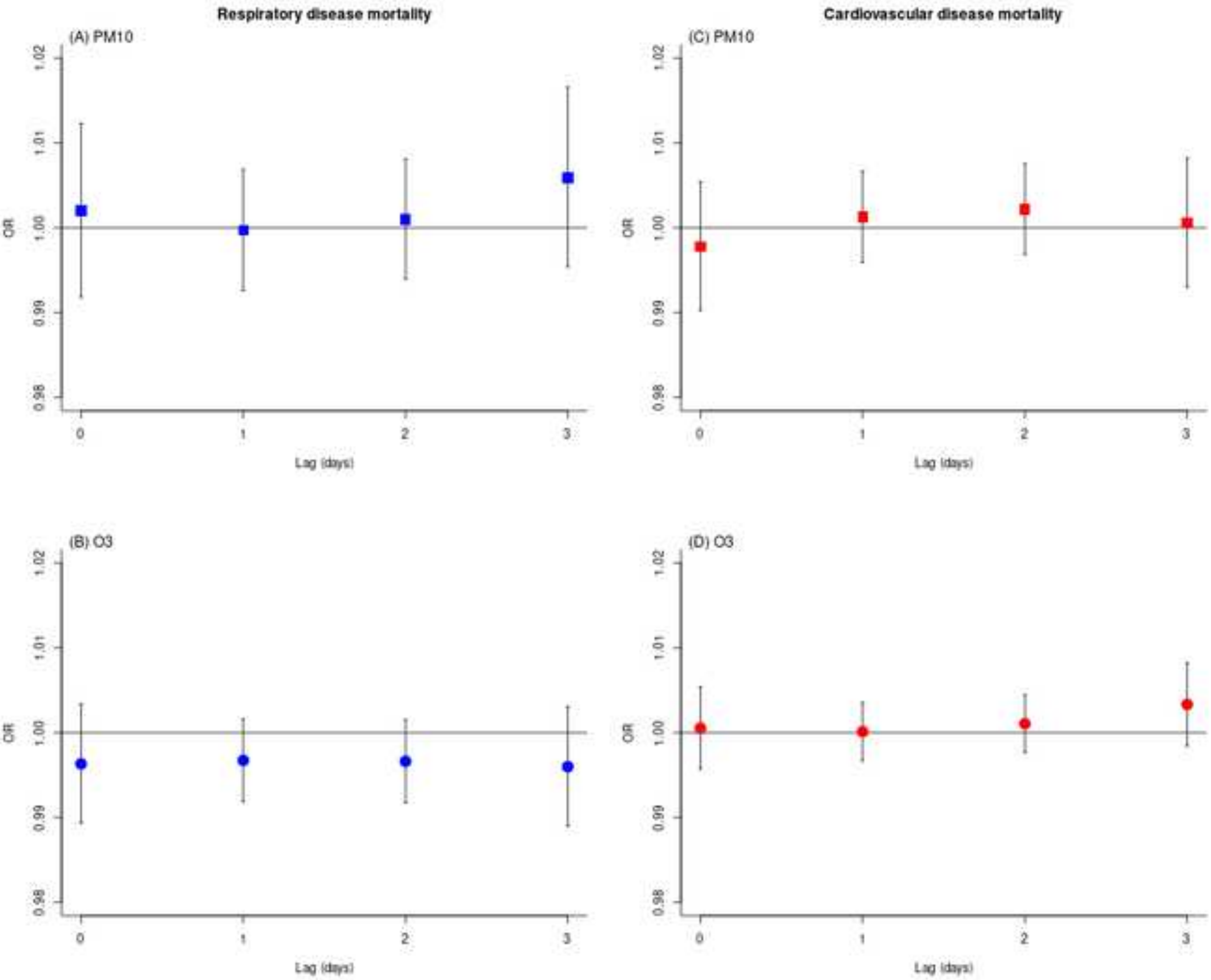

Click here to access/download
Supporting Information
S1 Appendix.pdf

Click here to access/download
Supporting Information
S1 Table.pdf

Click here to access/download
Supporting Information
S2 Table.pdf

Click here to access/download
Supporting Information
S3 Table.pdf

Click here to access/download
Supporting Information
S4 Table.pdf

Click here to access/download
Supporting Information
S5 Table.pdf

Click here to access/download
Supporting Information
S6 Table.pdf
