# Supplementary material for: Short-term association between ambient air pollution and cardio-respiratory mortality in Rio de Janeiro, Brazil
Source: PLoS One. 2023 Feb 16;18(2):e0281499. doi: 10.1371/journal.pone.0281499 (PMC9934392; doi:10.1371/journal.pone.0281499)
Supplement: Attachment — Submitted filename: Response to Reviewers.docx [file pone.0281499.s011.docx]

***Editor:***

*I am unable to make changes to authorship. Please add the new author as requested on 12/1/2022, in the location of your choosing and provide appropriate contact information, as needed. Then, resubmit the final version. Thank you for addressing all of my and the reviewers' concerns.*

***Additional Editor comments:***

*Thank you for the revisions to your manuscript. All requests have been adequately addressed. Please add the additional co-author with appropriate contact or other information as requested on 12/1 by the corresponding author and resubmit a final version.*

**Authors' response:** We thank you for your time and suggestions and for providing us with the opportunity to submit a final version of our manuscript. We added the new author with appropriate contact information. In addition, we have made few minor modifications to the manuscript, primarily the correction of typographical errors (lines 114, 205, 300) and the addition of citations for a few statements (line 264).

***Journal Requirements:***

*Please review your reference list to ensure that it is complete and correct. If you have cited papers that have been retracted, please include the rationale for doing so in the manuscript text, or remove these references and replace them with relevant current references. Any changes to the reference list should be mentioned in the rebuttal letter that accompanies your revised manuscript. If you need to cite a retracted article, indicate the article’s retracted status in the References list and also include a citation and full reference for the retraction notice.*

**Authors' response:** We checked the reference list and found no retractions. However, we corrected the manuscript title of reference number 34, which was incomplete.
